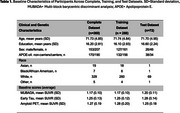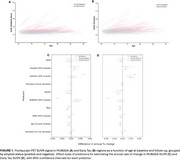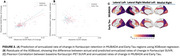# Predicting longitudinal accumulation of tau pathology in pre‐clinical population using machine learning: Data from the A4 and LEARN Studies

**DOI:** 10.1002/alz70856_107218

**Published:** 2026-01-08

**Authors:** Melika Saadati, Elizabeth Nemeti, Saima Rathore

**Affiliations:** ^1^ Hamedan University of Technology, Hamedan, Hamedan, Iran (Islamic Republic of); ^2^ Emory University, Atlanta, GA, USA

## Abstract

**Background:**

The preclinical stages of Alzheimer's disease (AD) represent a critical window, where pathophysiological changes like amyloid and tau accumulation begin years before clinical symptoms emerge. Accurately predicting the longitudinal trajectory of AD during these stages is essential for early intervention, enabling timely therapeutic strategies to slow or prevent progression and improve patient outcomes. Leveraging longitudinal data from the A4 and LEARN studies, we aim to evaluate the effectiveness of Flortaucipir‐PET imaging, along with clinical and genomic information, in forecasting the rate of change in tau pathology through machine learning models.

**Method:**

Participants (*N* = 360) were characterized using clinical (age, amyloid status, sex), genetic (APOE‐ε4), and imaging biomarkers (regional Flortaucipir‐PET standardized uptake volume ratio [SUVR] in MUBADA and Early Tau regions) (Table 1). We trained XGBoost regression models in a 4:1 split train‐test configuration to derive a prognostic index that forecasts individualized annualized rate of change in MUBADA and Early Tau SUVR. Model performance was evaluated using R‐squared (R^2^) scores.

**Result:**

The XGBoost models demonstrated strong predictive performance for MUBADA SUVR (R^2^=0.66), and moderate predictive performance for Early Tau SUVR (R^2^=0.42) (Figure 2A‐B). Among the predictors, amyloid status showed a significant association with changes in both Early Tau SUVR (*p* = 0.0004) and MUBADA SUVR (*p* = 0.0005), highlighting its critical role in tau pathology progression (Figure 1C‐D). Age also emerged as a significant predictor for changes in Early Tau SUVR (*p* = 0.0112) and MUBADA SUVR (*p* = 0.0141). These results underscore the importance of integrating amyloid burden and age into machine learning models to forecast individual trajectories of tau accumulation.

**Conclusion:**

This research presents a strong method for predicting disease advancement in preclinical AD, utilizing longitudinal data from the A4 and LEARN studies, machine learning techniques, and biomarkers. The findings indicate that Flortaucipir‐PET SUVR, when integrated with clinical and genetic information, provides considerable predictive capability for tau pathology. These results support the utilization of machine learning models for participant categorization, clinical trial enhancement, and individualized disease monitoring.